# Distance-dependent patterns of molecular divergences in tuatara mitogenomes

**DOI:** 10.1038/srep08703

**Published:** 2015-03-03

**Authors:** Sankar Subramanian, Elmira Mohandesan, Craig D. Millar, David M. Lambert

**Affiliations:** 1 Enviromental Futures Research Institute, Griffith University, Nathan 4111, Australia; 2 Allan Wilson Centre for Molecular Ecology and Evolution, Massey University, New Zealand; 3 Allan Wilson Centre for Molecular Ecology and Evolution, School of Biological Sciences, University of Auckland, Private 92019, Auckland, New Zealand

## Abstract

Population genetic models predict that populations that are geographically close to each other are expected to be genetically more similar to each other compared to those that are widely separate. However the patterns of relationships between geographic distance and molecular divergences at neutral and constrained regions of the genome are unclear. We attempted to clarify this relationship by sequencing complete mitochondrial genomes of the relic species Tuatara (*Sphenodon punctatus*) from ten offshore islands of New Zealand. We observed a positive relationship that showed a proportional increase in the neutral diversity at synonymous sites (dS), with increasing geographical distance. In contrast we showed that diversity at evolutionarily constrained sites (dC) was elevated in the case of comparisons involving closely located populations. Conversely diversity was reduced in the case of comparisons between distantly located populations. These patterns were confirmed by a significant negative relationship between the ratio of dC/dS and geographic distance. The observed high dC/dS could be explained by the abundance of deleterious mutations in comparisons involving closely located populations, due to the recent population divergence times. Since distantly related populations were separated over long periods of time, deleterious mutations might have been removed by purifying selection.

Tuatara are significant in terms of global biodiversity and the evolutionary history of reptiles^1^. They are the sister taxon to the Squamata (a group that includes lizards, and snakes)^2^ and are regarded as the most distinctive surviving reptilian genus. Although they resemble many lizard species, tuatara are not lizards at all. Instead, they are actually the last remaining member of a distinct order Sphenodontia, which has diverged from Squamata (all other reptiles) about 220 million years^3^,^4^. This unique reptile was once widespread on the mainland of New Zealand before the arrival of humans. It was thought to have been driven to extinction by exotic mammals introduced around 800 years ago^5^,^6^. Tuatara are now exclusively found on only 32 offshore islands around New Zealand.

It is well known that genetic similarity decreases with increasing distances between the habitats of individuals or population^7^. This is due to the balance between genetic drift and migration (gene flow). While migration typically introduces genetic variability, drift almost inevitably reduces it. However in the case of populations of a land vertebrate living on islands, the above relationship will largely be determined by genetic drift as the effects of migration is minimum. In the present study we examined this relationship using mitochondrial genomes of tuatara living in ten distinct islands that are 1–631 km separated. Although previous studies examined the relationship between geographic and genetic distances, their data were largely confined to neutral markers^8^,^9^,^10^,^11^. It is unclear how purifying selection modulates the genetic diversities at constrained regions of genomes. To examine this we estimated diversities at neutral and constrained sites of tuatara mitochondrial genomes.

## Results

**Figure 1:**
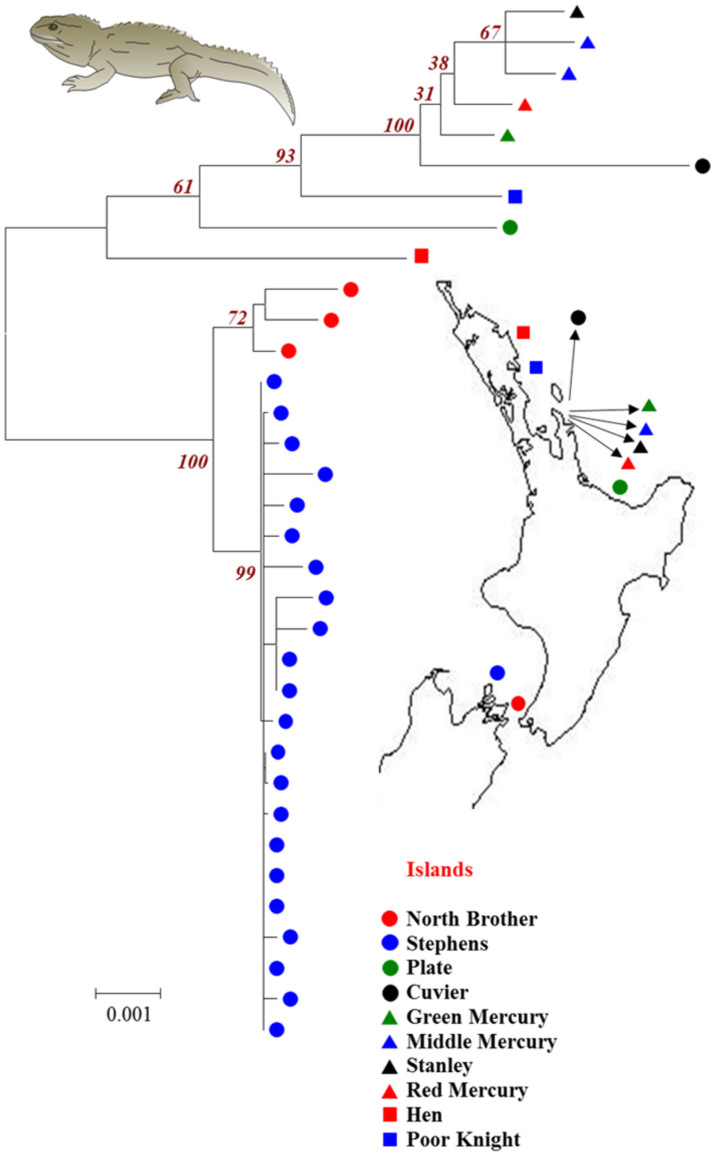
A maximum likelihood tree showing the phylogenetic relationships among tuatara from ten offshore islands of New Zealand. The tree was constructed using the complete mitogenome sequences. The bootstrap support (1000 replications) for major nodes are indicated. The map shows the approximate locations of the samples used in the study. The map and the image of tuatara were manually drawn by us using the software Canvas (ACD systems international Inc.) and Adobe illustrator (Adobe Systems Inc.).

Although tuatara live in 32 islands their population in most of the islands are very small ranging from tens to few hundreds with an exception of Stephens Island, which harbours >30,000 individuals (http://www.doc.govt.nz/documents/science-and-technical/TSRP47.pdf). As a result of fragmented and small population sizes tuatara are a highly endangered reptile and therefore obtaining samples is difficult. We managed to obtain tuatara samples from ten different islands. We sequenced the complete mitochondrial genomes of a single tuatara sample from seven islands, two from Middle mercury, three from North Brother and 22 from Stephens Islands (which has the largest population). To examine the phylogenetic relationships among tuatara from ten distinct islands, a maximum likelihood tree was constructed using complete mitochondrial genome sequences (Figure 1). We also obtained similar topology for the ML trees constructed using constrained and neutral sites (Figures S1 and S2 respectively – Supplementary material). For this purpose we used the diversities at synonymous sites (dS) and constrained sites (dC) respectively. The constrained sites consist of concatenated alignments of nonsynonymous sites of 12 protein-coding genes (gene NADH dehydrogenase subunit 5 is absent in tuatara^2^), rRNAs and tRNAs (see Methods).

Since tuatara are the only surviving member of the order Sphenodontia there is no outgroup species available to root the tree. This is because the divergence time between tuatara and its closest relative, the Squamata reptiles is ~220 million years^3^,^4^. However we did use the genome sequence of a chameleon (*Chameleo chameleon* - a Squamata reptile) and reconstructed the ML tree using only the constrained sites. As shown in Figures S3 and S4 (supplementary material) the topology is similar to the tree shown in Figure 1.

**Figure 2:**
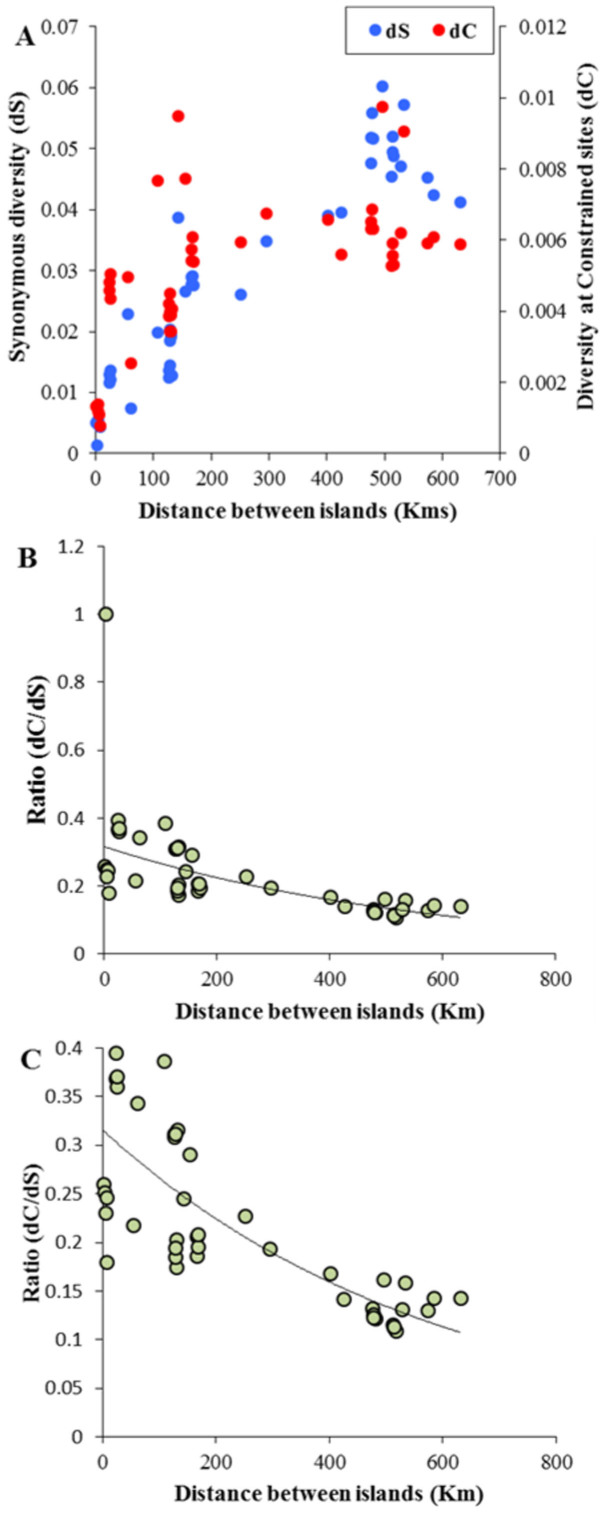
(A) Relationship between geographic distance and diversities at synonymous (Spearman *ρ* = 0.71; *P* < 0.001) and constrained (Spearman *ρ* = 0.92; *P* < 0.001) sites including the nonsynonymous sites of 12 protein-coding genes, tRNAs and rRNAs. The relationships were highly significant based on Mantel's test (B) Correlation between geographic distance and the ratio of constrained- to synonymous sites (dC/dS) (Spearman *ρ* = 0.82; *P* = 0.001). (C) Same as Figure 2B but the outlier is not shown. (The outlier was not removed and it was included for calculating correlation coefficient and for determining the statistical significance. Y-axis was rescaled to exclude the outlier in order to focus on the other data points).

Our samples are from a wide geographic range, including locations on off-shore islands in the North Island and Cook Straight of New Zealand. While the shortest distance between two islands was 1.3 km (Green and Middle Mercury Islands) the longest was 631 km between Poor knight and Stephens Islands (Figure 1). We examined the amount of diversity at neutral and constrained sites that could be explained by the distance between the islands. We plotted pair-wise diversities (dC and dS) between tuatara from different islands against the geographical distance between them. Figure 2A shows positive relationships between geographic distance with dC (Spearman *ρ* = 0.71) and dS (*ρ* = 0.92). These were highly significant using Mantel's test (*P* < 0.001). However a closer examination revealed that the relationship of dS with geographic distance is approximately linear and that of dC is non-linear. While dS estimates varied between 0.001 to 0.06 substitutions per site (s/s), dC range was much smaller between 0.001–0.01 s/s. However in Figure 2A we overlaid the relative diversities of dC and dS for each pair-wise comparison on the Y and Z axes respectively. This revealed that dC values estimated for pairs of individuals from closely located islands (eg. Stanley and Middle Mercury) were much higher than their corresponding dS. In contrast dC observed for pairs of tuatara individuals living in distantly separated islands (eg. Cuvier and Stephen) were relatively less than their corresponding dS. To demonstrate this clearly we computed the ratio of dC/dS for all pair-wise comparisons and plotted them against the geographic distances (Figure 2B). This resulted in a negative correlation (Spearman *ρ* = −0.82) that was highly significant (*P* = 0.001). The declining trend of dC/dS could be seen clearly after rescaling the Y-axis to exclude the outlier in order to focus other data points (Figure 2C) (The outlier was not removed and it was included for calculating correlation coefficient and for determining the statistical significance). The mean dC/dS ratio estimated for the comparison involving tuatara living in islands that were separated by <100 km was 0.35. In contrast, this ratio was only 0.13 for the pairs living in islands that are >500 km apart, which was 2.7 times smaller than that of the former. We also estimated the net diversities between populations by subtracting the intra-specific diversity from inter-specific diversities (see Methods). The correlations between the geographical distance and net diversities were also highly significant. The observed positive correlations between geographical distances and net-dC (Spearman *ρ* = 0.56) as well as between geographic distance and net-dS (Spearman *ρ* = 0.91) were highly significant (*P* < 0.001) using Mantel's test. A highly significant negative relationship (Spearman *ρ* = −0.74, *P* = 0.001) was observed between geographic distance and the ratio of net-diversities at constrained and neutral sites (dC/dS).

## Discussion

**Figure 3:**
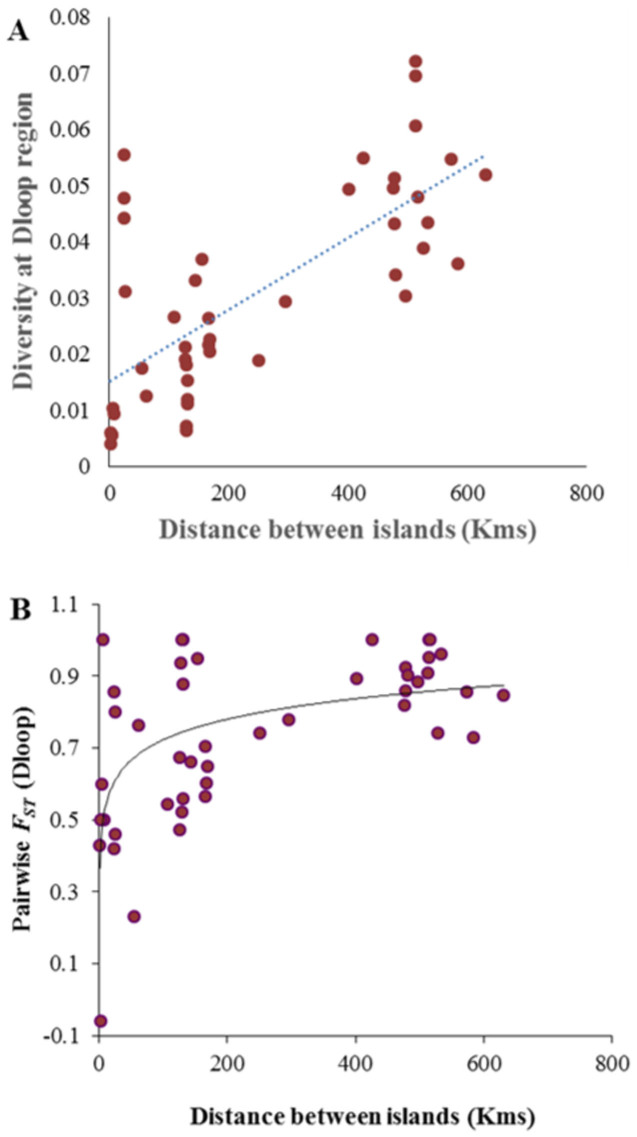
(A) Relationship between geographic distance and diversity at the D-loop region of tuatara mitogenomes (Spearman *ρ* = 0.68; *P* = 0.001). (B) Correlation between geographic distance and *F_ST_* estimated for populations from a pair of islands (Spearman *ρ* = 0.54; *P* = 0.004). The relationships were highly significant based on Mantel's test. Best fitting regression lines are shown.

The highly significant positive relationship between dS and geographic distance illustrated in this study suggests the possibility that neutral diversity increases proportionally with increasing distance between tuatara populations. We also examined the strength of the above relationship using the mitochondrial D-loop region, which was available for 58 individuals from the ten islands (2–11 individuals from each island) used in this study^12^. The results of this analysis also revealed a significant positive correlation between the geographical distance and genetic diversity at D-loop (Spearman *ρ* = 0.68; *P* = 0.001) (Figure 3A). This suggests that the correlation analyses reported in this study using complete mitogenomes are robust.

We also estimated the fixation index (*F*_ST_) between tuatara populations in order to examine the relative contributions of drift and migration (gene flow) using the D-loop data. The mean inter-population D-loop diversity was estimated to be 0.03. In contrast, the average intra-population diversity was only 0.005. The resulting *F*_ST_ was 0.85, which suggests a much smaller proportion of within population diversity. This suggests that between-(island)population diversities estimated in this study were not significantly affected by intra-population diversities. To further examine this we correlated pairwise *F*_ST _estimated for populations from different islands with the geographic distance between the islands. We observed a highly significant positive relationship (Spearman *ρ* = 0.54; *P* = 0.004) suggesting increase in population structure corresponds with the increase in the geographic distance between the island populations (Figure 3B). Since the mitogenome data from multiple individuals was not available for seven island populations we could not perform this analysis using the genome data. However the *F*_ST_ analysis from D-loop data imply that the relationship between genetic and geographic distances appear to be largely determined by genetic drift. This also suggests that migration rates of tuatara populations between islands might be minimal.

The positive relationship between geographic distance and neutral diversity suggests a direct relationship between geographic distance and time of divergence between the island populations. Further evidence for this prediction comes from our dC analysis. It is well known that at short timescales deleterious mutations are expected to segregate in populations due to the power of genetic drift. However they are selected against over time and are eventually eliminated from a population^13^,^14^. Previous studies using intraspecific comparisons of human mitochondrial genomes observed 5−10 times higher proportion of deleterious variations compared to inter-species divergence^15^,^16^. In this study we observed a high dC/dS for the comparisons involving tuatara living in proximal islands and much lower dC/dS values for the pairs of individuals inhabiting distal islands. The different dC/dS ratios actually imply the difference in the divergence times between tuatara populations. The observed higher dN/dS ratio could also be due to the effects of population sizes as small populations harbour more slightly deleterious mutations. However tuatara population sizes of nine of the islands examined in this study are extremely small (<1000) except for that in the Stephens island (~30,000) (http://www.doc.govt.nz/documents/science-and-technical/TSRP47.pdf) and hence any significant effects of population size on our results is unlikely. The higher dC/dS ratios (1.0) observed might also suggest the possibility of positive selection. However it is well known that the effect of genetic drift is much higher in small populations, which reduces the efficacy of natural selection^13^,^14^. Therefore the observed relationships cannot be explained by positive selection as the population sizes of tuatara living in most of the islands are small.

Studies of population genetics, evolution and ecology routinely examine the relationship between genetic and geographic distances in order to infer the demographic history of populations. However many studies use genetic data to estimate population genetic parameters without distinguishing whether the sequences are under selective constraints or evolve neutrally. Here we have shown that these two types of genomic regions behave quite differently. This suggests that future studies need to test their sequence data for neutrality to obtain unbiased relationship between genetic and geographic distances.

The temporal pattern of deleterious non-synonymous polymorphisms has been demonstrated by a number of previous studies^15^,^17^,^18^,^19^. Here we identified (geographic) distance-dependent patterns of divergences using island populations of tuatara. Our results suggest that the geographic distance that separates two populations (or closely related species) of land vertebrates can be used to quantify the relative time of divergence between them particularly when the dispersal or migration rate is minimum.

## Methods

Blood samples were collected from 34 tuatara individuals from ten islands around New Zealand (number of samples in parenthesis): Poor Knights (1), Cuvier (1), Stanley (1), Green Mercury (1), Plate (1), Hen (1), Red Mercury (1), Middle Mercury (2), North Brother (3) and Stephens Islands (22) (Fig 1). DNA was extracted from blood samples^20^ and the entire mitochondrial genome of each tuatara individual was amplified in 16 overlapping fragments each approximately 1–2 kb in length. The sequencing was done on two independent PCRs. One PCR product was sequenced in the forward direction and the other one in reverse. In cases, in which there was a SNP in one direction but the second sequence was not long enough to cover that SNP, or showing a different SNP, then the third PCR was performed. The complete tuatara mitochondrial genomes were obtained using long-range PCR of 1–2 kb DNA fragments. The Long-Range PCR carried out in a 20 μl volume containing 1X PCR buffer (Invitrogen), 50 mM MgCl2 (Invitrogen), 1 mg/ml BSA (Invitrogen), 250 μM mix dNTPs (BioLine), 0.50 μM forward primer (Invitrogen), 0.50 μM reverse primer (Invitrogen), 5 U Elongase Taq and 1 μl DNA template. The PCR mixes were amplified using an iCyclerTM Thermal cycler (BIO- RAD, USA). The amplification programme consisted of initial denaturing at 94°C for 4 min followed by 10 cycles consists of 94°C for 30 sec, 57.5°C (primer specific) for 30 sec, 8 min extension at 68°C and another 30 cycles consists of 94°C for 30 sec, 57.5°C (primer specific) for 30 sec and 68°C for 8 min. For the following 30 cycles the extension time was increased by 20 sec after cycle one. The final extension at 68°C for 4 min was followed. The PCR products were stored at 4°C for further analysis. The PCR products were subjected to electrophoresis in 1.5% agarose, stained with ethidium bromide (50 ng/ml) and visualized over ultra violet (UV) light.

The list of primers used are given in the Supplementary material (Tables S1–S3). For primer design and optimization the program primer 3.0 (http://frodo.wi.mit.edu/cgi-bin/primer3/primer3_www.cgi) was used. For limiting the DNA amplification to the long fragments and avoiding the amplification of shorter sequences, particular PCR conditions were used. This resulted in competition between the templates in each cycle in favor of the long template from the preceding cycle. After purification each fragment was sequenced in both directions using forward and reverse primers using an ABI 3730 sequence analyzer. This was important to verify (any) nucleotide variants. A third PCR was performed in the case of any inconsistencies. All methods were carried out in accordance with the approved guidelines of Griffith University, Nathan, Australia. All experimental protocols were approved by Griffith University, Nathan, Australia.

The complete mitochondrial genomes belonging to 34 tuatara individuals along with the reference (NC 004815) genome were aligned using the program *SeaView*^21^. The reference genome was used to identify the boundaries of protein-coding and noncoding sequences. To identify the most appropriate evolutionary models and other parameters, we used the program MODELTEST^22^ as implemented in MEGA^23^. Based on Bayesian Information Criterion (BIC) we found the model Tamura-Nei + Gamma best described the data. Using this model a maximum likelihood tree was constructed using MEGA. To obtain statistical support for each node we used the bootstrap resampling procedure with 1000 replications.

To estimate pair-wise diversities at synonymous sites (dS) we used third codon positions (3182 bp) or four-fold degenerate sites (1161 bp). Although both produced almost identical results we used the former for all analysis due to its size. For constrained sites (dC) we concatenated nonsynonymous (first two codon positions) sites of protein-coding genes, all tRNAs and two rRNAs (10360 bp in total). To estimate genetic diversities within and between populations we used HKY + Gamma model for constrained and synonymous sites using the alpha values of 0.05 and 0.11 respectively. This model was chosen using the program MODELTEST and it corrects for multiple substitutions, accounts for base compositional bias as well as corrects for rate variation among sites. To estimate the net diversity between populations we used Nei and Li's method *d_A_* = *d_XY_* − (*d_X_* + *d_Y_*)/2, where *d_XY_* is inter-population diversity and *d_X_*and *d_Y_* are intra-population diversities^24^. From a previous study^12^ we obtained 58 D-loop sequences of tuatara living in Green Mercury (3), Middle Mercury (2), Red Mercury (2), Stanley (3), Cuvier (4), Plate (6), Hen&Chickens (12), Poor Knights (11), Stephens (8) and North Brother islands (7). We excluded the flanking tRNAs and used only the D-loop region (928 bp) spanning between positions 95 to 1022 of the sequences reported by the previous study^12^. To estimate diversity we used TN92 + Gamma model using a shape parameter of 0.05.

Geographic distances between individuals from pairs of islands were obtained from the online resource *DistanceFromTo* (http://www.distancefromto.net/). To test the significance of any correlation between geographical distance and genetic divergence we used the program *Isolation by distance*^25^. Specifically we performed a Mantel test that is based on Spearman correlation coefficient, as some of the relationships reported here were nonlinear. However the strength of the relationship and the levels of statistical significance obtained using the Mantel test based on Pearson correlation coefficient (employed in the program *Genepop*^26^) were similar. We have reported the correlation coefficients and significance for the power law (log-log) relationships. However the significance observed for linear-linear or log-linear relationships were also highly significant (at least *P* < 0.007). To estimate pairwise *F*_ST_ between tuatara populations we used the software *DNASP*^27^.

## Author Contributions

S.S. and D.L. conceived the project. E.M. performed sequencing and all other laboratory analyses. C.D.M. contributed to sample collection and laboratory analyses. S.S. conducted data analysis and hypothesis testing. S.S. wrote the paper with inputs from all authors. D.L. and C.D.M. edited the paper.

## Supplementary Material

Supplementary Information Supplementary information

## References

[b1] Cree A. & Butler D. Tuatara recovery plan (New Zealand Department of Conservation, Wellington, 1993).

[b2] Rest J. S. *et al.* Molecular systematics of primary reptilian lineages and the tuatara mitochondrial genome. Mol Phylogenet Evol 29, 289–297 (2003). 13678684 10.1016/s1055-7903(03)00108-8

[b3] Benton M. J. Vertebrate Paleontology (2nd Edition). (Backwell Science, 2000).

[b4] Carroll R. L. Vertebrate Paleontogy and Evolution. (W.H. Freeman & Co., 1988).

[b5] Duncan R. P., Blackburn T. M. & Worthy T. H. Prehistoric bird extinctions and human hunting. Proc Biol Sci 269, 517–521, 10.1098/rspb.2001.1918 (2002). 11886645 PMC1690920

[b6] King M. The penguin history of New Zealand. (Penguin Books, 2003).

[b7] Wright S. Isolation by Distance. Genetics 28, 114–138 (1943). 17247074 10.1093/genetics/28.2.114 PMC1209196

[b8] Li J. Z. *et al.* Worldwide human relationships inferred from genome-wide patterns of variation. Science 319, 1100–1104, 10.1126/science.1153717 (2008). 18292342

[b9] Maes G. E. & Volckaert F. A. M. Clinal genetic variation and isolation by distance in the European eel Anguilla anguilla (L.). Biol J Linn Soc 77, 509–521.

[b10] Pistis G. *et al.* High differentiation among eight villages in a secluded area of Sardinia revealed by genome-wide high density SNPs analysis. PloS one 4, e4654, 10.1371/journal.pone.0004654 (2009). 19247500 PMC2646134

[b11] Xing J. *et al.* Genetic diversity in India and the inference of Eurasian population expansion. Genome biol 11, R113, 10.1186/gb-2010-11-11-r113 (2010). 21106085 PMC3156952

[b12] Hay J. M., Sarre S. D., Lambert D. M., Allendorf F. W. & Daugherty C. H. Genetic diversity and taxonomy: a reassessment of species designation in tuatara (Sphenodon: Reptilia). Conserv Genet 11, 1063–1081 (2010).

[b13] Kimura M. The neutral theory of molecular evolution. (Cambridge University press, 1983).

[b14] Ohta T. The nearly neutral theory of molecular evolution. Annu Rev Ecol Syst 23, 263–286 (1992).

[b15] Hasegawa M., Cao Y. & Yang Z. Preponderance of slightly deleterious polymorphism in mitochondrial DNA: nonsynonymous/synonymous rate ratio is much higher within species than between species. Mol Biol Evol 15, 1499–1505 (1998). 12572613 10.1093/oxfordjournals.molbev.a025877

[b16] Parsons T. J. *et al.* A high observed substitution rate in the human mitochondrial DNA control region. Nat Genet 15, 363–368, 10.1038/ng0497-363 (1997). 9090380

[b17] Ho S. Y., Phillips M. J., Cooper A. & Drummond A. J. Time dependency of molecular rate estimates and systematic overestimation of recent divergence times. Mol Biol Evol 22, 1561–1568, 10.1093/molbev/msi145 (2005). 15814826

[b18] Subramanian S. Temporal trails of natural selection in human mitogenomes. Mol Biol and Evol 26, 715–717, 10.1093/molbev/msp005 (2009). 19150805

[b19] Subramanian S. *et al.* High mitogenomic evolutionary rates and time dependency. Trends Genet 25, 482–486, 10.1016/j.tig.2009.09.005 (2009). 19836098

[b20] Sambrook J. & Russell D. W. Molecular cloning: a laboratory manual. (Cold Spring Harbor Laboratory).

[b21] Gouy M., Guindon S. & Gascuel O. SeaView version 4: A multiplatform graphical user interface for sequence alignment and phylogenetic tree building. Mol Biol Evol 27, 221–224, 10.1093/molbev/msp259 (2010). 19854763

[b22] Posada D. & Crandall K. A. MODELTEST: testing the model of DNA substitution. Bioinformatics 14, 817–818 (1998). 9918953 10.1093/bioinformatics/14.9.817

[b23] Tamura K. *et al.* MEGA5: molecular evolutionary genetics analysis using maximum likelihood, evolutionary distance, and maximum parsimony methods. Mol Biol Evol 28, 2731–2739, 10.1093/molbev/msr121 (2011). 21546353 PMC3203626

[b24] Nei M. & Kumar S. Molecular evolution and phylogenetics. (Oxford University Press, 2000).

[b25] Jensen J. L., Bohonak A. J. & Kelley S. T. Isolation by distance, web service. BMC genet 6, 13, 10.1186/1471-2156-6-13 (2005). 15760479 PMC1079815

[b26] Raymond M. & Rousset F. Genepop (Version-1.2) - Population-Genetics Software for Exact Tests and Ecumenicism. J Hered 86, 248–249 (1995).

[b27] Librado P. & Rozas J. DnaSP v5: a software for comprehensive analysis of DNA polymorphism data. Bioinformatics 25, 1451–1452, 10.1093/bioinformatics/btp187 (2009). 19346325

