# Supplementary material for: Distance-dependent patterns of molecular divergences in tuatara mitogenomes
Source: Sci Rep. 2015 Mar 3;5:8703. doi: 10.1038/srep08703 (PMC4346810; doi:10.1038/srep08703)
Supplement: Supplementary Information [file srep08703-s1.pdf]

Distance-dependent patterns of molecular divergences in tuatara mitogenomes

Sankar Subramanian, Elmira Mohandesan, Craig D. Millar and David M.Lambert

Supplementary material

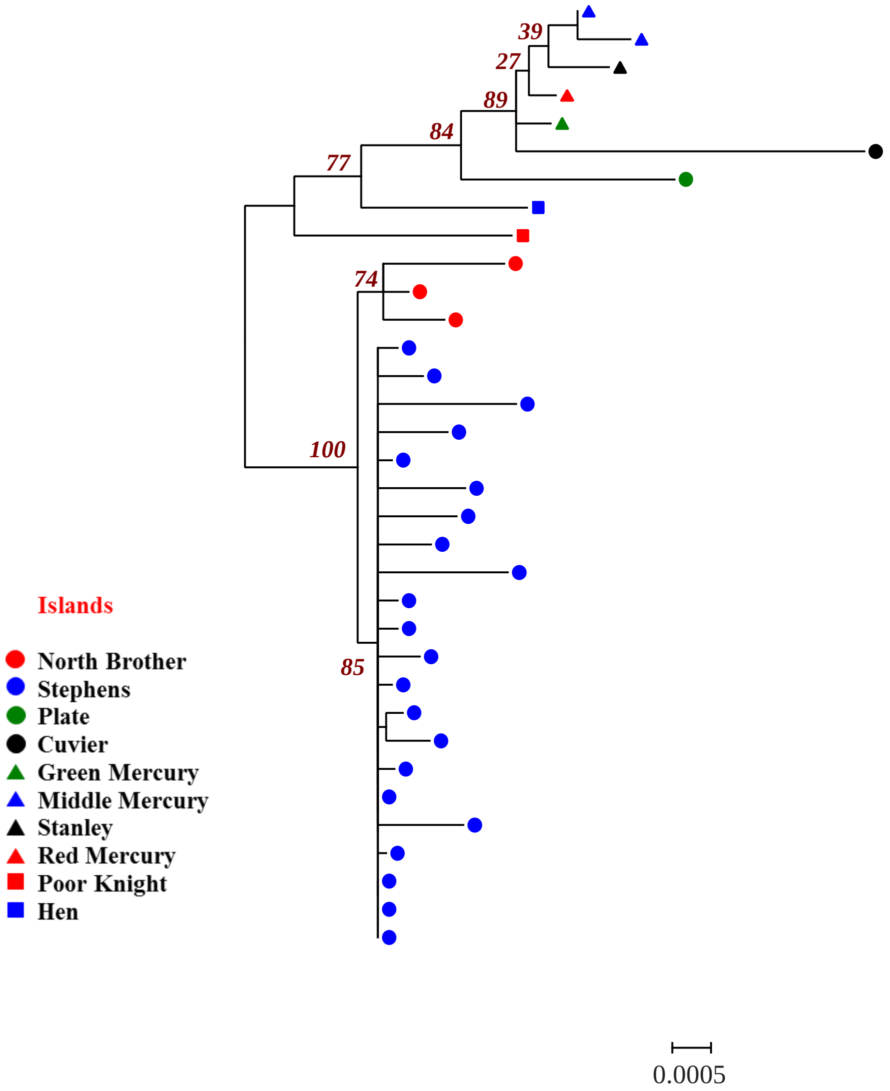

Figure S1. A maximum likelihood tree showing the phylogenetic relationships among tuatara from ten offshore islands of New Zealand. The tree was constructed using constrained sites of tuatara mitogenomes, which include nonsynoymous sites of 12 protein-coding genes, tRNAs and rRNAs. . The bootstrap support (1000 replications) for major nodes are indicated.

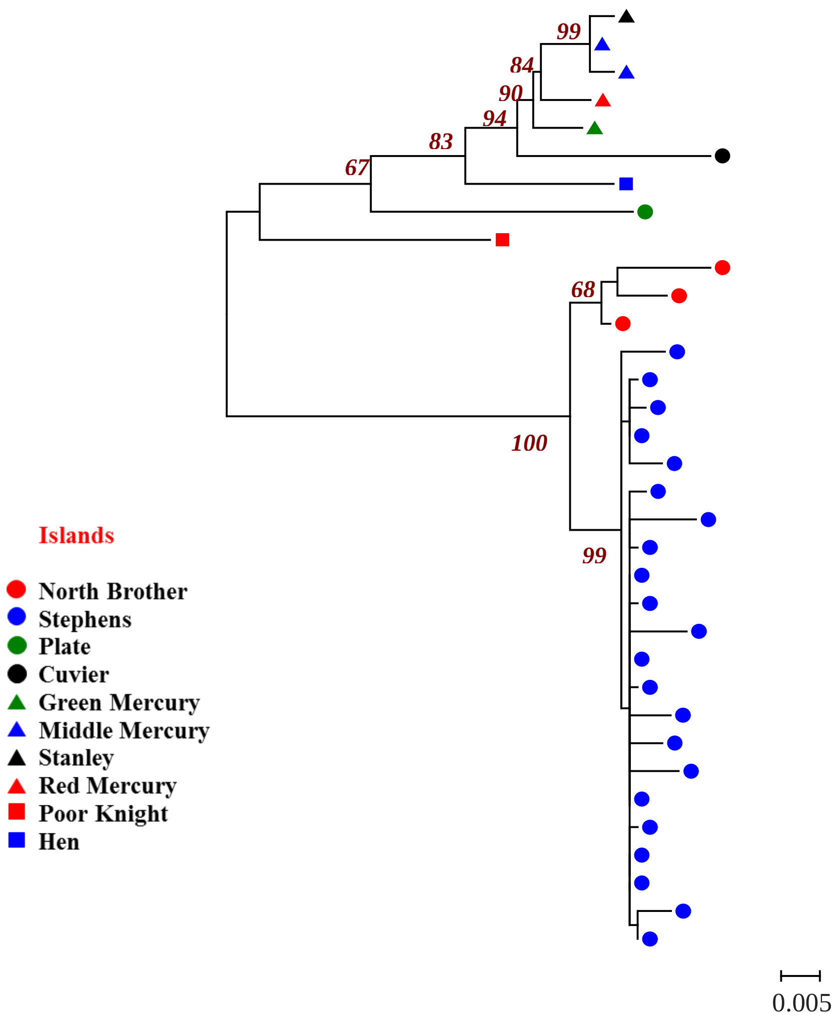

Figure S2. A maximum likelihood tree showing the phylogenetic relationships among tuatara from ten offshore islands of New Zealand. The tree was constructed using synonymous sites of tuatara mitogenomes. The bootstrap support (1000 replications) for major nodes are indicated.

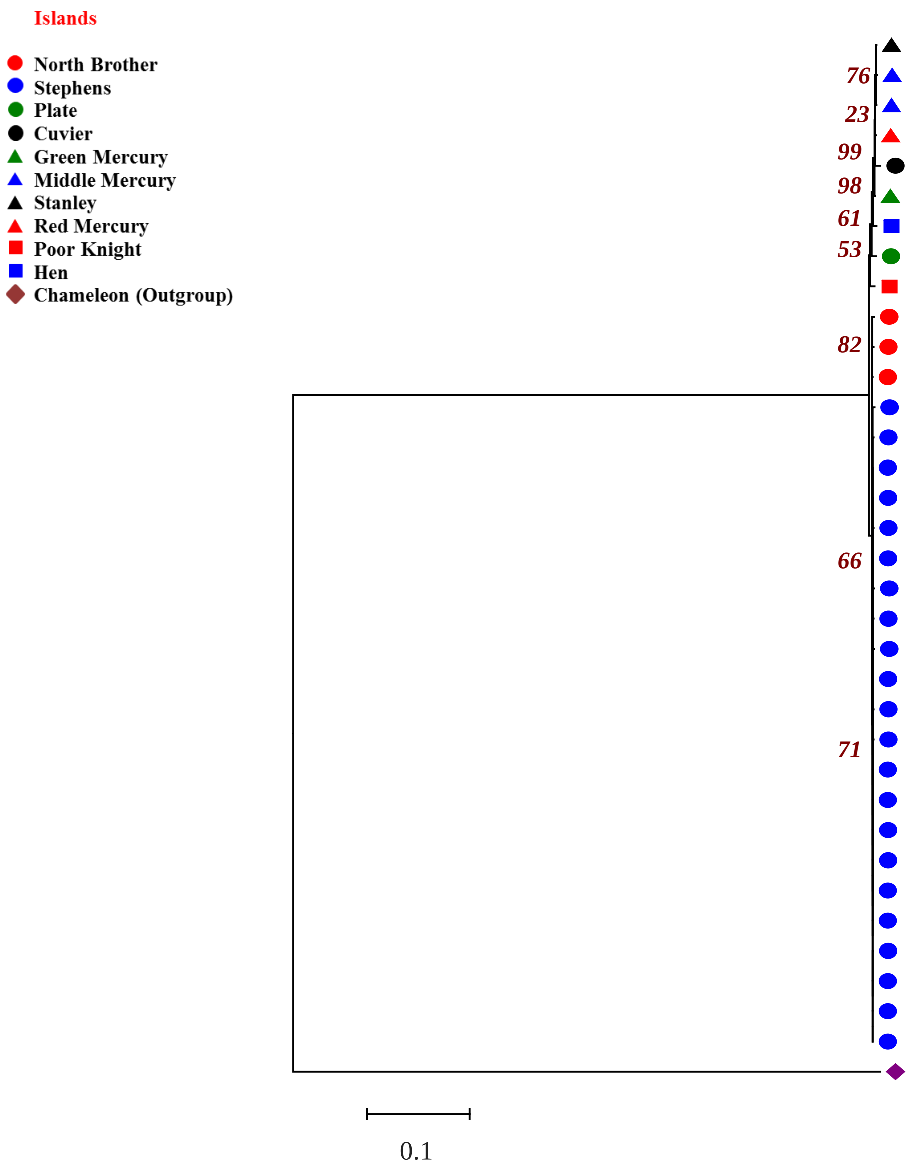

Figure S3. A maximum likelihood tree showing the phylogenetic relationships among tuatara from ten offshore islands of New Zealand and an outgroup (Chameleon - *Chameleo chameleon)* . The tree was constructed using the complete mitogenome sequences. The bootstrap support (1000 replications) for major nodes are indicated.

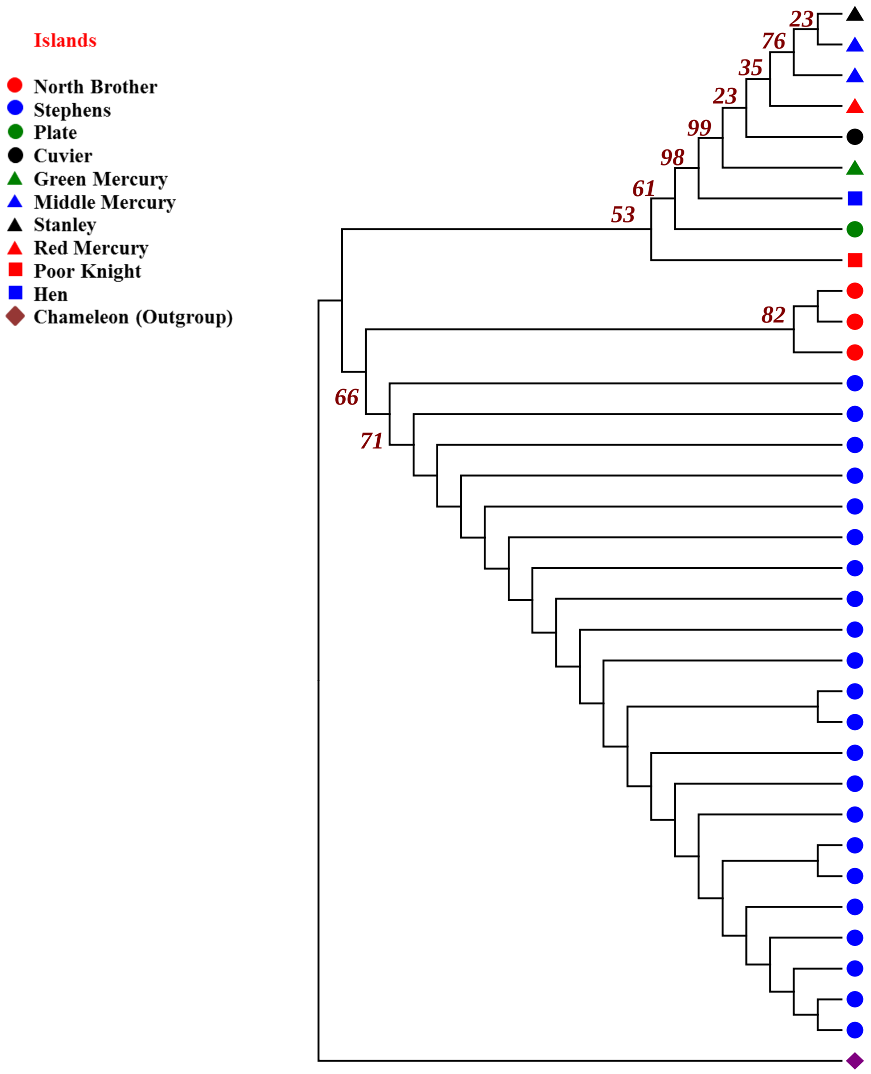

Figure S4. This is the same tree shown in figure S3, but only the topology is shown to clearly visualize the phylogenetic relationship.

Oligonucleotide primers:

Table S1: The external primers (5’ to 3’ direction) designed for amplifying the complete tuatara genome (set A)

Primer Name		Primer Sequence	
F01	GGCGTAGCAAAACCTGGATA	F09	AACTAGGCTTGGCCCCATT
R01	GCATATGGGCGTACTGGTTC	R09	CCTATATGGGCGATTGAGGA
F17	CCTTATTCATTTGATCCGTCCT	F25	TTGGCCACCAATGATACTGA
R17	GGTTCTTTCTTGTTTGAGTAGTCG	R25	TGGCAGGTGTGTTGAGATTG
F33	CATGGCCTATTACAGGAGCAA	F41	AACAACCTAAATCTCCTTCAATGC
R33	TGGGGGATAGGCTAGAGTGA	R41	GGGAGTTTTGGCTTGCTAGT
F49	TGATCTGGCACGTATTAACCA	F57	CGAAACCATTCGAAAACACC
R49	TTTGTTGGGTCTGGGTTTTC	R57	CTCGGGTTGCATGAAGGTAT
F65	CGCGCATAATATCCTATTCCA	F73	TCAAGTTGAAAAAGGCTCTGG
R65	GTCCGTTACACACCCATGC	R73	CCTGGGGTTCTTTTCTATCTCC
F81	CCGAAAACCTCATTTAGACCA	F11	TAGCCCTAACCCTGCTTTCA
R81	GATTGCTCCGGTCTGAACTC	R11	TTTGTGGGAGTAAAATGTGGTG
F03	CAATCTTATGATCGGGCTGA	F27	GACTGATCATGGGCCACAAC
R03	AATGGGGCTCGGTTTGTT	R27	GCACATGGTTGTGGGATAAA
F19	ATTATTGCCATCCCAACAGG	F43	ATTCGAAGCAACGCTTATCC
R19	AGGGGGAATCAATGGGTTAG	R43	ACATGCACCTCACAGAAAGAAA
F35	GGGGTAACAGTCACCTGAGC	F59	AAACCTCTTGTCCGCCATAC
R35	CCCAAAATGGTGGTCTATGG	R59	GTGCTCCTAGCAGGTCCTTG
F51	CCAACACCAACCAAAAATCC	F75	TACCCCGAAATCAAGTGAGC
R51	GAGGTCGAGAAGGAATGCAC	R75	TTAAGGCCTACGGTTTGGTG
F67	CCCCCACGAACATAGATTAGC	F13	TTTCTGAGGAAACCTGAAAGATT
R67	TRCATGGTGTGGTTGTRGGT	R13	TCAGCGTTTGTATGGCAAAG
F05	AAATCCAGGGCAAGGAATAA	F29	CGGTGATAATACCAGCAATGG
R05	GTGATCCTTTATTCAGGCACAT	R29	AGGCTAGGGCCATGGTTAAT
F21	CGCACTAACATTCATCGGAGT	F45	CAATGGTCTTAGCCGCAATC
R21	GGGGGTGTCCATGTAGTCAT	R45	TCCCGTTAGGCTTCATTCTG
F37	TTCCAATCATTAAGTTCTAGACAAC C	F61	GAAAATTTCACCCCAGCAAA
R37	GCGGTAGGGGTGTTAGATTG	R61	TGGGGGTTCTACTGGTTGTC
F53	CGCGCATAATATCCTATTCCA	F77	AAAGATTTCTCCGTGCATCC
R53	GTCCGTTACACACCCATGC	R77	TGTTCCGAGTTCCTTCTGGT
F69	TGCCTCAATGACACAAAGATT	F15	CCGAGGAGAATTAAGCTATCCA
R69	GCTGGCACGAAATTGACC	R15	CGGGTATACGGTTCATCCTG
F07	CTAATGCACCCCTCCATCAC	F31	ATTTCCTCCCAGAAGGTTCC
R07	TCTCATTGGGCAGTTTGTCA	R31	AGGCTTGAATAAGTGCTACTGC
F23	CAAGCTAGTTGAGCAACCACA	F47	TGAACGTATTAACAATCGAACACT
R23	GGCGTCTGTTGCGTTAATGT	R47	GGATGCCAGGTGGTTAGAGA
F39	GACATGAACTTCCCTAATTTTAACC	F63	TCTCGCTCAAGAAAAGAGGA
R39	CCAAGCATAATAAAGCGGAGA	R63	AYTCGTGAGGTCGAGAAGGA
F55	CCCCCACGAACATAGATTAGC	F79	CCGTGCAAAGGTAGCGTAAT
R55	CCCCCACGAACATAGATTAGC	R79	CCAAAACAGAGGGGGTCATT
F71	TTGGGGCCAAGATAAAATTG	F81	CCGAAAACCTCATTTAGACCA
R71	GCGGTAATGCTGTTTGACCT	R81	GATTGCTCCGGTCTGAACTC

Table S2: The external primers (5' to 3' direction) designed for amplifying the complete tuatara genome (set B)

Primer Name		Primer Sequence	
F02	CAACCTATTGCAGATGGCCTA	F10	GGTGAGGAGGAATGAACCAA
R02	GTTTGTGCTACCGCTCGAAT	R10	TCCTGTTATGGGTGGTAGGC
F18	TCTTACCTGGATTTGGGATAATCT	F26	TTGGTATCAGCAGAAGATGTCC
R18	TGCTTCTCATTTAATTATTCCTCCA	R26	CTAAAAGGCTGGTGCTGCAT
F34	TTTGTTTATTGTCTCCGAGGTG	F42	AGATCACATTTCATCCCCACT
R34	CCGATTTTCGTTTCCCTTCT	R42	TTAAGGCGTTCTGGTTGGTT
F50	CAACTGTCTCTTCCCCAACC	F58	CCGCAATATACATGCAAACG
R50	GGGGTGTTAGGGCTTTTGAT	R58	GGTTAGTGTGGCGTTGTCAAT
F66	TCCTTTCAACCACATATCCAG	F74	AACTAGCCCGCTACCCAGAT
R66	TTRACTCTTGGTTTAGGGGTTT	R74	TTGCCACAGAGCCGAATTAT
F82	TGTTCAACGATTAACAGTCCTACG	F12	ACCACTCCACCAAACACCTC
R82	GGCGAGAGGAGTTGAACCT	R12	GAGGCCTGCTCATCTAGGAA
F04	AACCCTAAACACCACGCAAG	F28	CAATAGTCCTAGTGTGGCTCTCA
R04	CTCGGACCCATAGGAATCAA	R28	GCCCTGTTACAAGGTGTTGG
F20	CCTTAGACATTATTCTCCACGACA	F44	CTGAACAACACCCTATTAATCACA
R20	TCATGTGGAGTATGCATCTGG	R44	TTATTGGTGTGAGCATGGTG
F36	TGCCTCCTACGACAAATCCT	F60	AGGATCGAACAACCCAACAG
R36	TGGTTGTGTGTTTGGGAGTC	R60	CAACACCCCTCCCATTTTATT
F52	GGYACGCCGCCTTATATT	F76	AAATAGGAAACAGCCTATACAAGAGG
R52	GGAAGATCCGTAAGCACTGG	R76	AATTATTGTGTAATGACCAGTAGGTTG
F68	AAGGAAGCCTTATGCATTTTT	F14	CAGGCCTGATAAGAAAAGGAA
F68	CACTGGTGTGCTGATACTTGC	R14	AAGCATGGGCTGTTACGATT
F06	CCTTTGACACACATCCATGC	F30	ACGCAAGGCCATAAATGAAC
R06	GCTTGTGGATAGGGCGATAA	R30	CTACCACAAGGGCTGGGATA
F22	CCTTTGAGAGGCTTTCTCTTCA	F46	TCATTTGCCTTCGACAAACA
R22	GCAATTTGTATGGGGTAGGC	R46	AAAGTCATCATGTGGAGAGAAGG
F38	CTCGCCTCCCATTTTCTATG	F62	CACCGCCCAATCAGTAAAAT
R38	GAACTAACTCTTCATTCGGCTCA	R62	TTTATTTAAAACTCCGGTTCTGG
F54	TCCTTTCAACCACATATCCAG	F78	AGGCATGCACCTAGGAAAGA
R54	TTRACTCTTGGTTTAGGGGTTT	R78	TTTCGCCAGGAGACAGTTG
F70	ACACCCCCAAGGGTACACAG	F16	TATTGACATCCGCCTGAACA
R70	GGGGATCTAATCCCAGTTTGA	R16	TCCTGCTGCTAGTACGGGTAA
F08	GCCGTAGAAGCCACCATAAA	F32	CCCATATGACCGGCTACAGT
R08	TAGTGGGGCCAGTTTTTGTC	R32	AAATCATACGGCGAGACCTG
F24	TCCGTGTTCTACATAATCACCA	F48	TTACAGGAGCTGGCGTAGTG
R24	GGAGTCAAATGAGAGGTCTTTGT	R48	AGAGTTGACTCGGGGTCAGT
F40	GCCTGTCATTACAACGCTCA	F64	GGYACGCCGCCTTATATT
R40	GGGAGAAGGAAAATTGTTGGA	R64	GGAAGATCCGTAAGCACTGG
F56	CGCTTTTAGAGGGGAGGAAC	F80	CCTTGTGCAAAAGCAAGGAT
R56	TACGTCGGCGGTGTAATGTA	R80	CCTGGGGTAACTTGGTTCAA
F72	TCGCCAGTCTACCTTGTGAA	F82	TGTTCAACGATTAACAGTCCTACG
F72	CAACCTATTGCAGATGGCCTA	R82	GGCGAGAGGAGTTGAACCT

Table S3 :The most used primers (5´ to 3´ direction) for amplification of tuatara mitochondrial genome, using long-range PCR.

Primer Pairs (F/R)	Product Size (bp)	Primer Pairs (F/R)	Product Size (bp)
F01/R05	1,064	F41/R47	1,298
F05/R10	1,208	F47/R52	1,243
F10/R15	1,274	F52/R57	1,191
F15/R20	1,240	F57/R62	1,202
F20/R25	1,165	F62/R67	1,210
F25/R31	1,304	F67/R72	1,257
F31/R36	1,242	F72/R77	1,156
F36/R41	1,160	F77/R02	1,287
F01/R21	4,121	F41/R63	4,296
F20/R43	4,403	F61/R02	4,535
